# Fundamental theory on multiple energy resources and related case studies

**DOI:** 10.1038/s41598-023-37653-5

**Published:** 2023-07-06

**Authors:** A. J. Jin

**Affiliations:** The Maritime Faculty, Ningbo University, Ningbo, 315000 Zhejiang Province China

**Keywords:** Engineering, Energy science and technology, Renewable energy

## Abstract

Herein, I methodically optimize a distributed energy resource in terms of the production, management, utilization, and/or transaction of renewable energies during the deployment process. I deliver a theoretical mathematical model that allows users to visualize three critical output functions of their energy preference, including output power, energy economy, and carbon footprint. The model delivers three eigenstates derived by a power utility matrix (PUM) model. PUM transforms three-input parameters (3i) into three-output functions (3o) through 3i3o-transformation. It is ubiquitous, and its systematic characterization is discussed. Moreover, I discover a mathematical conversion relationship translating energy generation to carbon emissions. Various case-studies demonstrate the optimal energy resource utilization. Furthermore, an energy blockchain approach is employed for microgrid design, development, and carbon reduction. Finally, the authors demonstrate the energy–matter conversion relationship that improves carbon emissions for energy production, reducing the beta factor of carbon emissions to 0.22 kg/kilowatt hour for carbon peak and to zero for carbon neutrality.

## Introduction

As a part of the industrial power goal to address emissions and climate change issues, the global scientific community has reached a consensus on the need to curb carbon emissions^1–3^. Scientists have dedicated great efforts for decades to both energy-efficient and carbon-free methods to address power industry needs. The field of distributed energy resources (DERs) has been very interesting and has gained considerable attention for its potential in helping reduce emissions^4–6^.

The Paris Agreement set goals to address climate change issues^1,2^, defined steps for governments and multiple technology sectors to achieve, and proposed ways to mitigate the currently large carbon emissions. Carbon greenhouse gas (GHG) emissions from energy production can lead to climate anomalies, and thus, there is an urgent need to reduce carbon emissions. Because GHGs increase solar irradiance absorption, which leads to rapid glacier melting and the disruption of fragile ecosystems^7^, a climate emergency has been declared^2^.

The desire to meet the carbon neutrality goal has resulted in unprecedented international collaboration between citizens, academics, industry leaders, and government officials. Ideally, all sources of electricity will be stable, economical, and environmentally friendly^5^. Over the past few decades, renewable energy (RE) technology that can definitively meet the world’s energy demands has been developed, such as solar photovoltaic (PV) energy, wind energy, ocean energy^8–13^, hydrogen fuel cells, and energy storage (ES) technologies^14–18^. For instance, governments have been able to achieve major reductions in carbon emissions across all major business sectors. California has introduced a series of energy-themed goals, policies, and programs. Carbon cap and trade initiatives have begun to gain traction as a system to lower economic barriers to carbon reduction measures^19^.

Recently, with rapid advances in commercialization, renewable energy technologies have been widely applied. Major commercial RE sources, including solar and wind power, are unstable or intermittent by nature. The best utilization of RE usually involves integrating various types of complementary power generation (PG), ES, and commercial grid power (GP). Researchers have innovated or advanced technology that enables customers to leverage the great value of RE sources. The Carbon Border Adjustment Mechanism (CBAM) is designed and abided by the European Union (EU) to put a fair price on the carbon emitted during the production of carbon intensive goods that are trading in the EU. It is critical for advanced renewable technologies to remain competitive when commercial rules for carbon pricing, such as CBAM, are met.

Carbon pricing scenarios include a wide range of low-cost and cost-saving options associated with high energy efficiency, schedule optimization, alternative energies, ES, and fuel switching, i.e., transitioning from less environmentally friendly energy sources to more RE sources. The recent exponential growth in energy consumption demand has resulted in an urgent need to identify RE sources that can meet this demand and be operationalized at large scales.

Many valuable commercial and technological advances in energy decarbonization have been achieved^20,21^. Despite the unforeseeable challenges of daily changes in solar power and wind power instability, many countries have developed advanced technologies that enable them to use RE sources^22–24^.

For those who cannot participate in public utilities with mainly RE grids, RE can include locally produced systems that form microgrids and integrate various types of complementary PG and storage processes^25,26^.

The energy generated in a microgrid can be monitored and distributed using meticulously derived and advanced algorithms^27^ that match the supply of energy producers with the demands of consumers, ensuring that power stability and quality are maintained.

In addition to producing and consuming energy, producers and consumers can trade surplus energy with other users and/or profit from energy-related transactions. Blockchain technology encourages data sharing and collaboration through the Internet of Things, which enables buyers and sellers to conduct such energy transactions in an easy and transparent manner^28,29^.

Regarding RE sources, it is necessary to study how these systems perform and evolve both over time and space. For example, a detailed investigation of alternative energy sources in Sweden revealed that solar irradiance and wind speed are negatively correlated with one another on both hourly and annual scales^30^. To optimize the stability of the energy output on a seasonal basis, it was recommended that this system should operate with 70% solar power and 30% wind power. The complementary outputs of solar PV and wind power should be verified in the partition on a case-by-case basis when designed for tandem use. The fluctuations in these two energy sources are much smaller than when each energy source is used separately. As microgrids become more popular and efficient than ever before, the need for large datasets related to microgrid consumption, transactions, and management will correspondingly increase.

Transactions between end-users and prosumers^31,32^ have been investigated by several research groups. For example, some researchers have explored data and machine learning^6^, while other researchers have published investigative results^33^ regarding energy blockchain (EBC) applications involving homes and buildings and for distributed peer-to-peer (P2P) energy trading. In this study, I proposed a smart algorithm for a distributed energy resource (DER) that employs the inputs of utilization data to help achieve the stated optimal goals. Prior researchers have achieved significant progress in this area^30,35–37^; for example, Zhang et al. provided valuable technology components for RE systems. Here, I investigate a general approach to obtain a novel and valuable fundamental framework that will lay the groundwork for determining the process exergy. A novel framework that advances our knowledge in this field and the present cause for RE applications is imperative.

Power utility matrix (PUM) methods^34^ provide a typical case of PUM design with a smart energy approach. The PUM is a key component for best use realizing a smart energy system.

In studies on extensive RE systems, there are several factors for system characterization. It is crucial to identify the best DER and/or energy management system for three kinds of critical outputs for any given microgrid: power output, smart energy use/transactions, and carbon footprint.

RE is becoming  a mainstream energy that contributes to the 20 TW of annual power required to entirely meet the world's current energy demand. Researchers have made significant progress in increasing our knowledge about RE and distributed energy resources^38–40^. This knowledge is applicable to the exploitation of the full use of RE capacity; for example, it is valuable to be able to fully benefit from the current installed solar-wind power capacity that can deliver 15%of total electricity in China. Based on the above knowledge, I proposed a novel mathematical framework to solve the challenge of multiple RE supplies. The multiple energy resource (MER) system can facilitate the smart energy of a smart energy system. An important application can be derived from the aforementioned mathematical framework. The energy–matter conversion relationship (EMCR) is inspired by the mass and energy conversion relationship and applied as an important concept^41^. In this article, the EMCR connects GHG production to energy production.

The focus in this article is to formulate a simple and ubiquitous framework of smart microgrids and energy blockchain. To achieve a stable power supply, the employment of the PUM model can reduce the power strain on the grid during peak demand periods. Additionally, users can employ commercially available control strategies such as load shedding, demand response, and energy storage to maintain a robust microgrid^42^.

Moreover, it is valuable to develop communication and networking technologies for smart microgrids and to leverage machine learning in smart microgrids^42^. Energy blockchain utilizes blockchain technology to manage energy transactions among producers, consumers, and microgrids. The decentralized system of EBC ensures secure and transparent transactions, making it an ideal solution for managing energy transactions between renewable energy producers and consumers, which is a very exciting research area^43^.

This article is Structured as follows. In Section I, I introduce the background and the urgent need for green and economical power resources. Then, REs and the PUM are introduced. In Section II, the mathematical model of the PUM and several applications are discussed. Furthermore, in Section III, the system characteristic matrix (K,ij) and systematic ways to solve PUM models with the PUM (K,ij) characteristics are discussed. In Section IV, the EBC approach is explored and studied. Finally, in Section VI, future work and the directions and valuable assets for solutions to meet carbon neutrality goals at a global scale are discussed.

## Background

### Characteristics of common renewable energy sources

It is important to establish a foundational framework to provide a background for the general mathematical description below. The energy optimization scheduling of a microgrid is a multiobjective optimization problem with multiple constraints. The maximal energy utilization efficiency, which is known as the exergy of the DER system, can be achieved and requires the proper scheduling of the DERs, ES, and power. The objective of the optimization is to achieve the maximum overall benefit through reasonable coordination, where all parties representing sources, storage, grids, and loads communicate to coordinate utilization scheduling. The microgrid can operate in either the grid-connected mode or the independent mode, both of which require proper scheduling of the DERs, ES, and load. For instance, a microgrid can operate more reliably under the independent operation mode. However, the scheduling process for DERs is complex. A mathematical model may describe a ubiquitous law, using the PUM model, as described below.

**Figure 1:**
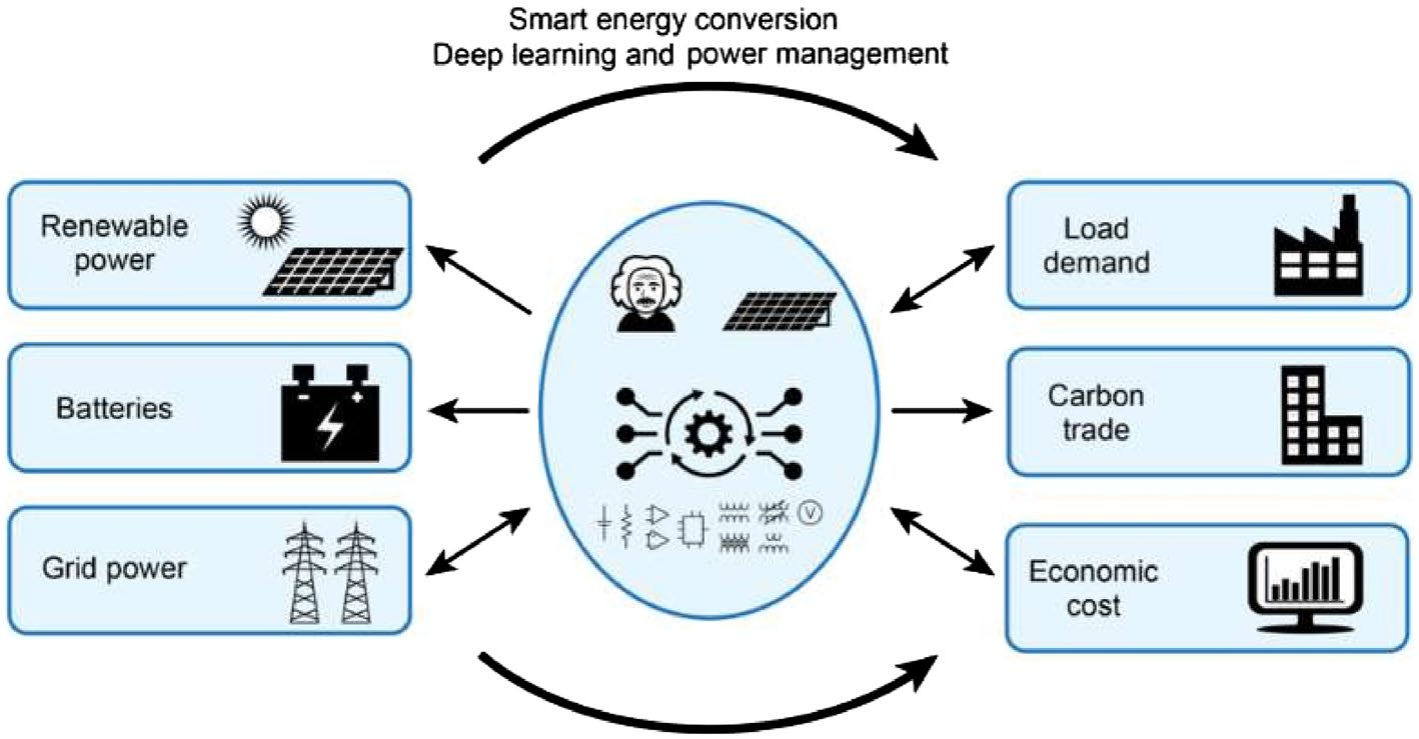
Detailed schematic of the power input elements and triple outputs. The middle column illustrates a working model and its solutions that render a smart power utility matrix (PUM) system.

Figure 1 illustrates a DER, microgrid, and simplified PUM model to address the power, cost, and carbon emissions of supplying energy. The left-hand side of Fig. 1 represents three inputs, namely PG, ES, and GP, which are large power resources. The right-hand side includes the power output, the user’s power economy, and carbon emission data. The focus of optimized energy scheduling is to achieve both economic and environmental goals while delivering the required power output.

A microgrid usually offers much better predictability and reliability in the long term when operated in the grid-connected mode than in the independent mode. The PUM model for a microgrid matrix is investigated to produce stable output power to meet demand and to meet certain economic and environmental goals in terms of capacity. The PUM model can be expressed as follows:

1 $$\left[ {\begin{array}{*{20}c} {{\text{P}}_{CP}^{t} } \\ {C_{ECO}^{t} } \\ {e_{CD}^{t} } \\ \end{array} } \right]\, = \,\left[ {\begin{array}{*{20}c} {K_{1\_PG} } & {K_{1\_ES} } & {K_{1\_EX} } \\ {K_{C\_PG} } & {K_{C\_ES} } & {K_{C\_EX} } \\ {K_{CD\_PG} } & {K_{CD\_ES} } & {K_{CD\_EX} } \\ \end{array} } \right]\, * \,\left[ {\begin{array}{*{20}c} {{\text{P}}_{PG}^{t} } \\ {C_{ES}^{t} } \\ {e_{GP}^{t} } \\ \end{array} } \right]\, * \,\tau ,$$

where $\tau \, = \,\frac{\Delta t}{T}$, ∆t is the scheduling time, and T is 1 h. For example, if the scheduling time is 10 min, $\uptau =1/6$.

Pt,CP denotes the power output. Ct,ECO denotes the financial value of the power consumption to its user. et,CD represents the carbon emissions derived from both the DER and the power utility.

Following these formulae, one may derive the value dependency relationship^44^. In the microgrid economic and carbon emission calculation model, the first row of the matrix indicates that the power demand of the load in a microgrid must be met in total by the current level of energy production, the available stored energy, and the energy provided by an external power grid. In other words, consumers are ensured that they can use electricity without service disruptions.

The typical value of each of the PUM coefficients in the first row typically ranges from 0.95 to 1. Therefore, each K,1j is simply assigned a value of 1, as follows:

2 $${\text{K}},{11}\, = \,{1};\,{\text{K}},{12}\, = \,{1};\,{\text{K}},{13}\, = \,{1}.$$

To maintain a specific power quality, the power balance constraint must be satisfied. Therefore, the first row of the PUM can be rewritten as follows:

3 $${P}_{CP}^{t}= {P}_{PG}^{t}+ {P}_{ES}^{t}+ {P}_{GP}^{t},$$

where ${P}_{CP}^{t}$ stands for the electricity power demand of consumers, ${P}_{PG}^{t}$ for the power generated by a generator, ${\mathrm{P}}_{\mathrm{ES}}^{\mathrm{t}}$ for the power exchanged by the ES systems, and ${\mathrm{P}}_{\mathrm{GP}}^{\mathrm{t}}$ for the power exchanged by the microgrids and power grids.

The net electricity cost is the sum of income and expenses. Income is generated when users purchase electricity from the microgrid and/or from the power grid. By managing peaks, valleys, and storage, it should be possible to maintain a balance between supply and demand without costly power spikes or deficits. Based on current technological development, I demonstrate scientific principles that may be universally applicable in the field. Each principle is an applicable tool for experts in the field to aid in smart power system design. The net electricity cost of operating a microgrid is shown in the second row of Eq. (1).

${K}_{C\_PG}$ is the cost coefficient of generating 1 kWh of electricity from a generator, which may include the equipment cost, depreciation, and operation maintenance.

The cost of operating an ES system is expressed as follows:

4 $$\begin{aligned} {\text{c}}_{{{\text{ECO}}_{{{\text{ES}}}} }}^{t} \, & = \,K_{{C_{ES} }} \, * \,\left( {P_{ES}^{t} \, * \,\tau } \right) \\ & = \,\left( {K_{Bat - opm} \, + \,K_{Bat - ll} \, + \,K_{Bat - el} } \right)\, * \,\left( {P_{ES}^{t} \, * \,\tau } \right), \\ \end{aligned}$$

where ${K}_{C\_ES}$ stands for the cost coefficient of charging and discharging 1 kWh of electricity from the ES system, which may include the equipment cost, depreciation, and operation maintenance; $K_{Bat - opm}$ for the operating and maintenance cost coefficient of charging and discharging 1 kWh of electricity from the ES system; $K_{Bat - ll} \,$ for the equipment depreciation cost coefficient of charging and discharging 1 kWh of electricity from the ES system; $K_{Bat - el}$ for the energy loss cost coefficient of charging and discharging 1 kWh of electricity from the ES system.

The income and the cost of purchasing energy from an external power grid are expressed as follows:

5 $${c}_{ECO\_EX}^{t} ={K}_{C\_EX}{* (P}_{GP}^{t}*\uptau ),$$

6 $$K_{C\_EX} \, = \,\left\{ {\begin{array}{*{20}c} {f_{{\text{s}}} } & {P_{GP}^{t} < 0} \\ {C_{{\text{b}}} } & {P_{GP}^{t} \ge 0} \\ \end{array} } \right.,$$

where ${f}_{s}$ is the price at which energy is sold to the power grid, and ${c}_{b}$ is the price at which electricity is purchased by the microgrid from the power grid.

The cumulative net electricity cost of operating a microgrid over a period $t_{n}$ is given as follows:

7 $$C_{ECO}^{t} \, = \,\sum\limits_{0}^{{t_{n} }} {C_{ECO}^{{t_{1} }} \, + \,C_{ECO}^{{t_{2} }} \, + \,C_{ECO}^{{t_{3} }} \, \ldots + \,C_{ECO}^{{t_{n} }} } .$$

### Values of typical K-characteristics in the transformation matrix

The commercial cost structure of current technology with solar RE (i.e., lithium battery) grid power is presented as follows^44^. At the current commercial stage, some K-values are provided as follows:

8 $$\begin{gathered} {\text{K2,1}}\,{ = }\,{0}{\text{.09 USD/kWh,}} \hfill \\ {\text{K2,2}}\,{ = }\,{0}{\text{.10 USD/kWh,}} \hfill \\ {\text{K2,3}}\,{ = }\,{0}{\text{.05 USD/kWh}}{.} \hfill \\ \end{gathered}$$

K2,1 is the cost of PV power per kWh, which mainly includes the depreciation of the purchase and installation cost of the PV modules, inverters, transformers, brackets, and power distribution equipment, as well as daily maintenance costs. The fuel cost of the PV modules is zero; the ratio of equipment depreciation and maintenance costs (including the operation cost) is generally approximately 7:3. The actual equipment lifetime is approximately 20 years. The largest variable affecting the depreciation cost is the local optical resources. The richer the optical resources are, the lower the investment equivalent to the unit of installation is, and the lower the depreciation is. The value of K2,1 is derived from the average level in China, and for regions with extremely rich optical resources, such as India and Pakistan, the Middle East, North Africa, and Central America, K2,1 may be halved with the same equipment.

K2,2 is the cost of energy storage, which mainly includes equipment depreciation and power loss. Maintenance costs are relatively low, mainly composed of personnel salaries, but solar/wind power distribution network energy storage power stations are generally maintained by solar/wind power station personnel part time. Under the condition of determining the technical conditions, the cost of energy storage is most affected by the utilization frequency of the energy storage system: a higher recycling frequency can reduce the apportioned depreciation cost of the latter two factors of equipment within a certain range, which is mainly due to the calendar and cycle lives of energy storage batteries. When any one of the calendar years or cycles reaches the design value, the energy storage equipment needs to be replaced; therefore, when the calendar life is reached before the cycle life, the increase in the frequency of use can proportionally reduce the depreciation and allocation, and vice versa, and the impact of reaching lifetime lmit is not significant. In addition, a higher recycling frequency can also reduce the power loss, which is mainly due to the internal thermal management, monitoring, and other supporting power consumption ratios of the energy storage system decreasing with increasing frequency of use. This K2,2 value is estimated based on the average charge and discharge frequency once a day.

K2,3 is the cost of the external purchase and sale of electricity. In general, the fs and Cb values are not equal during the trade, depending on whether the microgrid investor and the power grid company have signed a power purchase and sales agreement (generally, the absolute value of Cb will be equal to the absolute value of fs). The K2,3 value is the average of the market conditions of the previous two factors. In countries such as Germany and China, governments have implemented tariff policies over two decades that have provided substantial RE pricing incentives to increase RE sales on the grid.

As a result, the entire matrix is completed with estimates of a typical carbon emission case as follows. Energy production may vary. Thus, actual numbers depend, in part, on the procurement process within the industrial eco-chain.

1. The investigation leads to typical technical specifications at the current level.
2. The provided specifications based on technical data are extracted from commercially available products.

Furthermore, the typical values of all the PUM coefficients in the second row are as follows. The detailed model in the studies on K,2j shows time dependence. The third row of the matrix represents the carbon emissions generated by the operation of the microgrid^45,46^.

I selected typical values of CO2 emissions. For the current technological stage of development, some K-values in the specification are as follows:

9 $$\begin{aligned} {\text{PV}}:{\text{ K3}},{1}\, & = \, 0.0{\text{9283 kg}}/{\text{kWh}}, \\ {\text{WT}}:{\text{ K3}},{1}\, & = \, 0.0{\text{11128 kg}}/{\text{kWh}}, \\ {\text{Li}} - {\text{ES}}:{\text{ K3}},{2}\, & = \, 0.000{\text{57 kg}}/{\text{kWh}}, \\ {\text{Clean coal std}}.:{\text{ K3}},{3}\, & = \, 0.{\text{832 kg}}/{\text{kWh}}. \\ \end{aligned}$$

The amount of carbon released (in commercial DERs) is dependent on the maturity of the related technology, which may show substantial dependence on the time, manufacturing approach, operating conditions, and location in the supply chain.

## Power utility matrix for smart energy

### Linear integrative model of smart energy

In this study, a scientific description that may be universally applicable for engineers skilled in the smart grid field is provided. In accordance with Eq. (1), a more general mathematical equation is an integral equation. In another form of summarizing a typical overall PUM characterization, the PUM model (with the 3i3o model) and the PUM matrix are expressed as follows:

10 $$\left[ {\begin{array}{*{20}c} {En_{CP}^{T} } \\ {C_{ECO}^{T} } \\ {Cm_{CD}^{T} } \\ \end{array} } \right]\, = \,\int\limits_{{T_{0} }}^{T} {\left[ {\begin{array}{*{20}c} 1 & 1 & 1 \\ {K_{C\_PG} } & {K_{C\_ES} } & {K_{C\_GP} } \\ {K_{CD\_PG} } & {K_{CD\_ES} } & {K_{CD\_GP} } \\ \end{array} } \right]\, * \,\left[ {\begin{array}{*{20}c} {{\text{P}}_{PG}^{t} } \\ {P_{ES}^{t} } \\ {P_{GP}^{t} } \\ \end{array} } \right]} \, * {\text{dt}}{.}$$

The energy, cost, and carbon emissions are obtained from Eq. (10). The PUM values are obtained from Eqs. (2), (8), and (9).

The general problem becomes an interesting mathematical formulation that can be solved using the following framework. The linear algebraic equation may be simplified by solving for the three eigenvalues and deriving the three eigenstates in the eigenspace. The linear algebraic problem leads to characteristic (eigenvalue) equations in the eigenspace that have a diagonalizable matrix and three orthogonal variables. The 3 × 3 square matrix is a diagonalizable matrix. I can derive such eigenstates from linear algebraic calculations of the matrix as the determinant, minors, and cofactors.

### Simulation studies

Electricity from a microgrid can be transferred to users through a series of physical transactions; these transactions occur at both on-peak and off-peak times. There are many independent power producers and microgrids currently competing for customers in energy distribution networks.

Conducting power demand management (PDM) is imperative for a microgrid to ensure that it can meet the relevant energy demands during emergencies. Microgrid loads are generally classified as critical loads, controllable loads, or uncontrollable loads. Power systems must be able to meet the critical load requirement at any given moment.

### Dual energy storage: a working mode

In the case of an emergency, a controllable load can be cut off or adjusted as needed. Under normal circumstances, the purpose of optimizing the load use and energy savings may be to manage the response to demand. For example, transferable loads such as heat loads can be used when electricity prices are low and the system is not experiencing peak demand. The load in a given household is directly related to users’ electricity preferences and comfort level. Users can carry out oversight of their power needs and consumption through the use of smart devices (e.g., smart switches and smart thermostats). ES is one of the key constituents of a regular microgrid; dual ES (DES) is both useful in grid operation and valuable for the battery lifetime. RE, such as solar PV, has variable availability that is approximately one quarter of the time; its actual time is dependent on the location of its operation. To supply power continuously, the remaining power must be provided through complementarity, such as ES and batteries. The working of the ES is discussed below.

(i) The working caveats may be extended to a variety of applications. For example, I have designed an improvement in the utilization of ES.
(ii) ES is one of the critical components in a DER. Researchers have discovered that all ES batteries have optimal charge‒discharge cycle depths. Their battery lifetime depends on an important parameter: the depth of discharge of batteries (DoDb).

**Figure 2:**
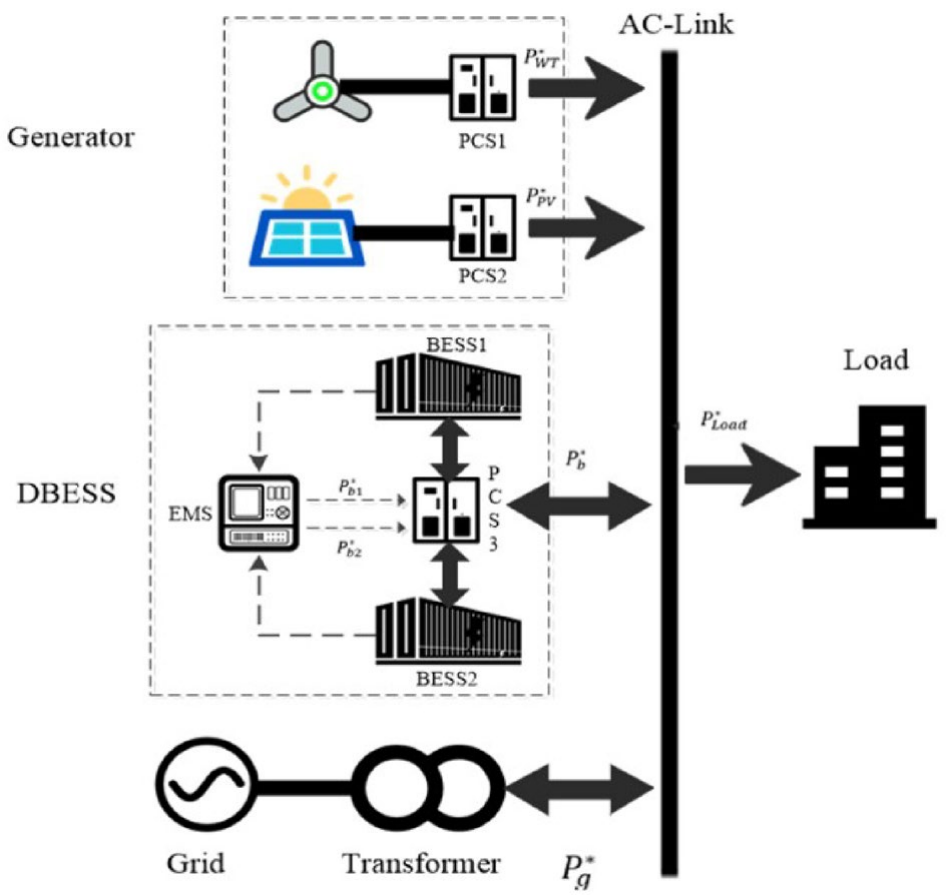
Microgrid architecture illustrating the dual energy storage (DES) mode.

The smart microgrid architecture is shown in Fig. 2 below. Each ES device is programmed in the DES mode; it recurrently works in a full cycle of charge and discharge. Deep cycles are very beneficial for the ES lifetime by removing numerous insufficient charge–discharge cycles.

### Simulations of PUM model with distributed energy-resources

**Figure 3:**
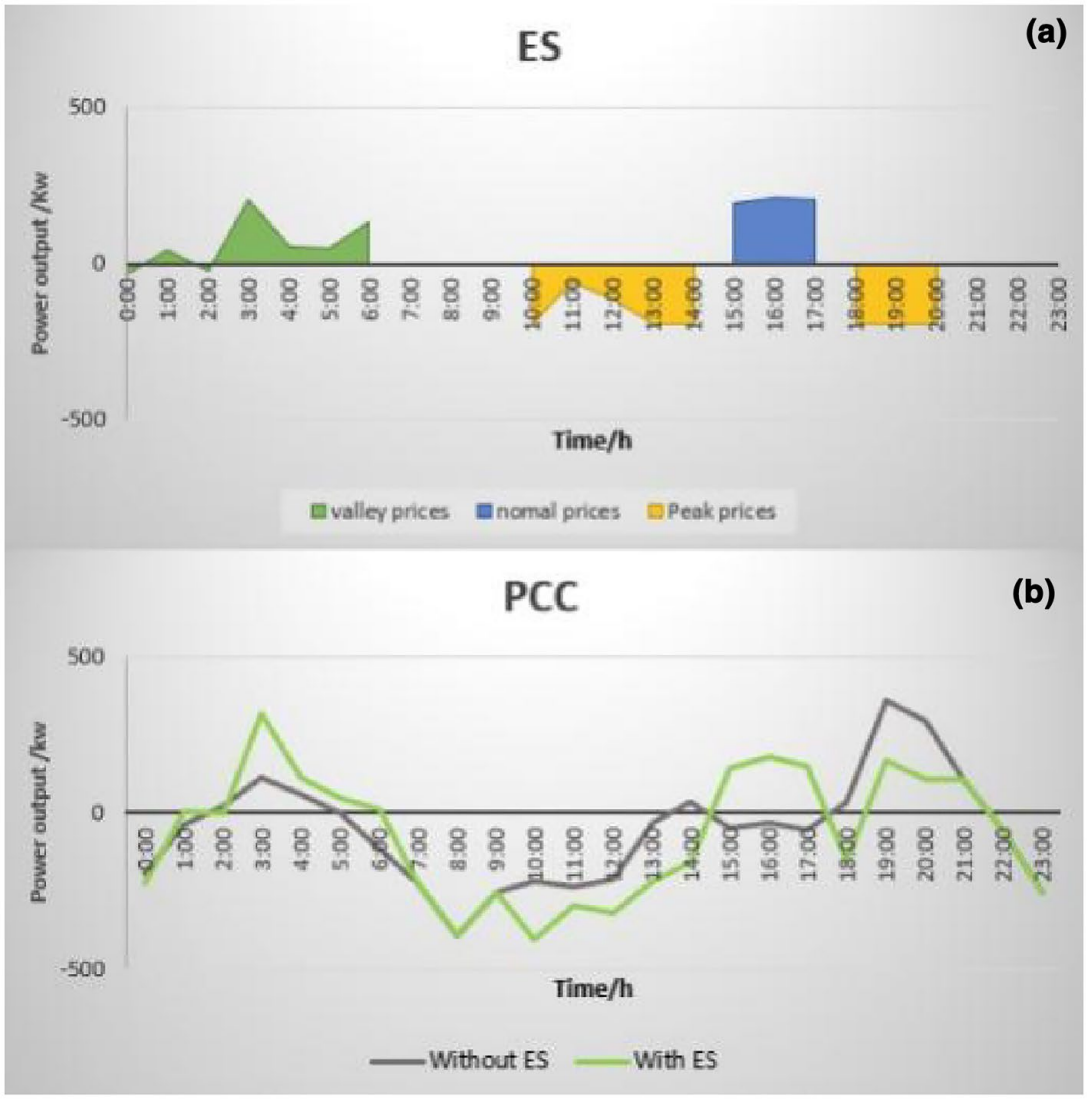
Typical energy in-and-out flow: (**a**) scheduling of distributed energy system with energy storage and (**b**) comparison at the point of common connection.

Figure 3 contains the test results for a simulation of a metro-transportation system. These test results demonstrate the typical energy in-and-out flow and are shown as follows: (a) scheduling of the distributed energy system with energy storage; (b) comparison at the point of common connection. In the DES system, DES can be used effectively to overcome the prediction error and to track the day-ahead trading plan of the microgrid system in real time.

A comparison of various methods is presented in Table 1. The table shows a list of the economic costs of the methods based on simulations once per day, under peak rotation, and with various hardware configurations.

**Table 1:** The power and cost data are compared for a few distributed power configurations in the table. Note: A negative cost indicates income, and a negative peak or valley load indicates output.

Methods	Base load	Traditional wind-solar	Smart wind-solar with energy storage
Cost (USD)	1.434.75	12.26	− 80.19
Peak load (KW)	6908	40	− 1289
Valley load (KWh)	2608	− 398	22

Note that Cost "-" means income; Peak or valley load "-" indicates output quantity.

**Figure 4:**
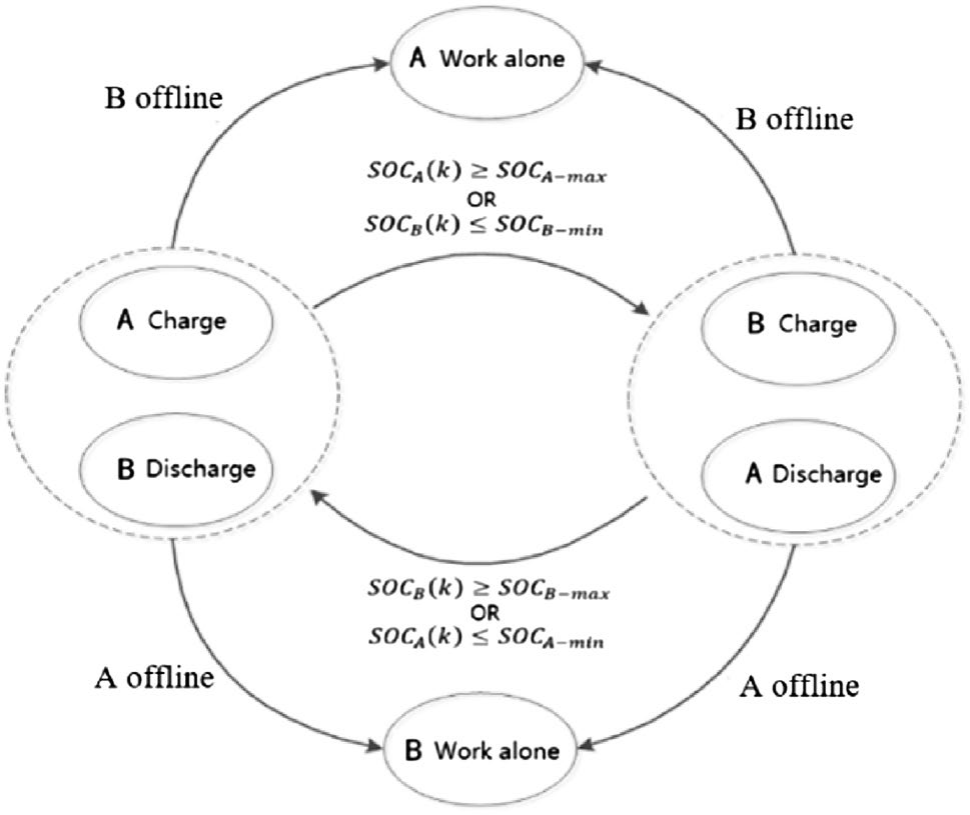
Schematic diagram of the charge‒discharge cycle showing the cooperative working mode for a DES system.

According to the analysis in the last section, the DES mode is utilized with benefits. The working schematic diagram is shown in Fig. 4, along with its benefits. The energy supply appears the same for the outside viewer, providing ES, increasing the storage lifetime, and  including many operational nuances for potential benefits.

### Microgrid system architecture and the transformation matrix

The mathematical model of the PUM can be readily applied to all microgrid systems. Equation (1) is rewritten as follows:

11 $$\left[ {{\text{Load}},{\text{ carbon}},{\text{ Fin}}} \right] \, = \, \left[ {{\text{K}},{\text{ij}};{\text{ 3X3}}} \right]*\left[ {{\text{PG}},{\text{ ES}},{\text{ GP}}} \right].$$

In the previous section, I deployed the PUM transformation matrix, which provides the output solutions based on the input DER variables. As a result, the derivation of the technically complex architecture of a DER becomes a simplified DER specification. The three-output functions (3o) in the simplified PUM design problem are as follows:

12 $$\left[ {{\text{3o}}} \right] \, = {\text{ PUM }}* \, \left[ {{\text{3i}}} \right],$$

where 3i represents the three input parameters and PUM is a 3 × 3 square matrix.

According to Wolfram et al.^47^, the solution to Eq. (12) is readily obtained in orthogonal space and can be characterized by a set of three distinct eigenstates with three eigenvalues: λ,_1_, λ,_2_, and λ,_3_. Thus, the details of this derivation are omitted here.

In the relevant eigenspace, each output function is specifically related to each particular eigenstate with the corresponding input variable, which is called the orthogonal variable. Each of the critical outputs of the three eigenstates depends on its orthogonal variable. For example, the increment in the power output in an eigenspace is achieved by tuning its orthogonal eigenvariable, which will not affect the value of the carbon emissions within a specified range.

The carbon index of the PUM system is derived as follows:

13 $$\lambda ,{\text{c }} = \lambda ,{2}/\lambda ,{1}.$$

The output function is built based on the critical values in the eigenstates within its critical variables in the eigenspace. The beta factor is the ratio of output energy and carbon emissions.

An artificial intelligence (AI) algorithm issues the command to set the output power values automatically. When the EBC is implemented, the power conversion will be connected to the RE and to the ES that has a point of common connection with an inverter/converter function.

A distributed energy approach is shown in the working diagram in Fig. 4 with a DES and in the 3i3o model illustrated in Fig. 1. The model can be expanded in applications in the case of a DES.

## Energy blockchain

In a decentralized energy system, energy supply contracts can be directly communicated between producers and consumers. Enabling an EBC can result in a considerable number of transactions between producers and consumers, which makes each transaction less expensive overall. Blockchains facilitate direct interactions and transactions between local energy producers and consumers by eliminating the need for a third-party monitoring platform.

 Software instructs the system connected to the output terminal, i.e., the client node. A ledger from the EBC may be implemented in 5 min upon request (or in a different agreed-upon set of time. There are several network blockchain options for energy-industry applications. Many researchers have discovered various scientific phenomena/data, and many studies have been reported^48–54^. Researchers have conducted investigations and have expanded the knowledge on this subject by referring to the results in the literature^55–59^.

**Figure 5:**
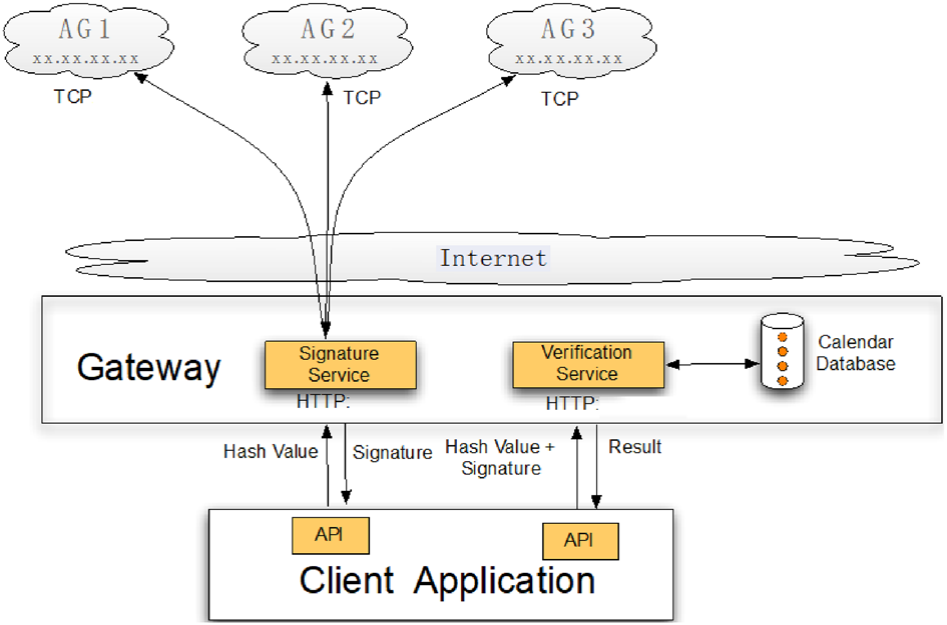
Flowchart of keyless blockchain-as-a-service interfaces.

The distributed energy resources can be traded with clients via the internet by choosing one of the blockchain options. The classic blockchain structure of EBC illustrates layers of provider-customer-clients and peer-to-peer gateway to the internet. For example, a smart grid can be applied for all digital electricity where the client nodes stand for all goods: AG1, AG2, AG3. Keyless blockchain-as-a-service interfaces (KBaaS) are presented in Fig. 5. Its advantage is trust and it meets the security needs established for both supply and demand groups. Figure 5 provides microgrid-management data provenance based on a lightweight and keyless blockchain -as-a-service (KBaaS). The blockchain structure of EBC is illustrated as follows.

## Discussion

The share of global electric generation is expected to reach a total of 20 TW soon. The shares of global RE generation should be increased: solar and wind power must reach 78% to achieve carbon neutrality^60^. To achieve the emission peak by 2025 and the carbon neutrality goals by 2050, the current policy calls for 47–78% RE in the primary energy supply. The world requires 20 TW to maintain quality of life, with approximately 78% accounted for by renewables. This corresponds to 6.2 and 9.4 TW of solar and wind power to achieve carbon neutrality.

I studied the beta factor, which is a metric defined as the rate of EMCR across multiple energy systems. It is 0.45 kg/kWh for traditional coal-fired power plants. However, it should be less than 0.22 kg/kWh to achieve the desired carbon emission peak; and it should be nearly zero to achieve carbon neutrality.

Researchers may be challenged to attain a pure categorical input (Cat). Each category may be composed of some combination of reated input entities. The current PUM in this article has advantages in deriving the eigenstates and eigenvalues because the PUM is a 3X3 square matrix. This PUM can deliver three eigenstates or eigenvalues. As a result, some simple metrics for carbon neutrality have emerged elegantly.

The current PUM utilized the essential classification of three input categories that may be expanded in every category as follows: Cat-1 has renewable energy resources; Cat-2 has energy storagies; and Cat-3 has microgrids. A remark is that the PUM model may encounter situations that exceed the basic PUM scheme in practice; each input parameter may be combination of multiple parameters, such as several renewable energy sources. Furthermore, customers may have more competing requests in an application while their knowledge of data and machine deep learning can be valuable for a good resolution. Each Cat may be properly bundled to meet the model requirements shown later.

To define the predicted RE power supplies as a single input in the PUM model, one would need to formulate Cat-1 as the single input. Moreover, the text at above has demonstrated an example of Cat-2 as illustrated in Fig. 2; the DES is managed in an algorithm that may be expanded in various applications. Furthermore, to formulate Cat-3 as the single input by supplier(s), I believe that the general categorical solution can be conveniently applied from the input power. In general, Cat-3 is provided by the power input, e.g., the grid provider. This input power may be composed of EBC and/or power supplier solutions of the microgrids. Our future work will collect data systematically across all the afore-mentioned categories so that the PUM model may be implemented for all types of smart microgrids.

In practice, one may handle complexity and employ a more complex system-solution^61^. The PUM model is an advantageous approach as an overall solution technique for microgrids. Several more complex systems are studied experimentally and methodically in a separate work^62^.

## Conclusions

In conclusion, I have presented a theoretical study toward developing a predictive model that in turn realized a mathematical PUM specified by a 3i3o square matrix. Every element in the 3 × 3 PUM matrix is important in that it contributes to the specification of the total distributed energy system. Moreover, the PUM forms orthogonal variables based on linear algebraic operations of the input parameters. For example, a rule-of-algebra can be applied in the eigenspace to tune the output power in a specified range without affecting the other two functions that include cost and/or carbon emissions. Moreover, an important discovery is the proposal of a beta factor to illustrate the ratio of carbon dioxide emissions and the total output power. The beta factor was less than 0.22 kg/kWh when a carbon emissions peak was achieved, and it was nearly zero for the carbon neutral system.

I conclude the knowledge of the power utility matrix in detail is crucial to resolve critical outputs for the designer's tools, to enlist critical output parameters, and to identify the fundamental mathematical model. The related knowledge is very important to determine the direction and provide guidance to effectively achieve carbon neutrality with REs. The above framework provides important guidance for DER design, construction, and operation.

## Data Availability

The data that support the findings of this study are available from Ningbo University, China; but restrictions apply to the availability of these data, which were used under licence for the current study, and so are not publicly available. Data are however available from us upon reasonable request and with permission from Ningbo University, China. The datasets used and/or analysed during the current study are available from the corresponding author on reasonable request.

## Abbreviations

RE: Renewable energies
DER: Distributed energy resource
3i: Three-input-parameters
3o: Three-input-functions
EBC: Energy blockchain
GHG: Greenhouse gas
PV: Solar photovoltaic
ES: Energy storage
PG: Power generation
GP: Grid power
CBAM: Carbon border adjustment mechanism
IoT: Internet of Things
P2P: Peer-to-peer
DER: Distributed energy resource
PUM: Power utility matrix
EMCR: Energy matter conversion relationship
MER: Multiple energy resources
PDM: Power demand management
DES: Dual ES
DoDb: Discharge of batteries
EMS: Energy management system
AI: Artificial intelligence
EV: Electric vehicle
PMU: Power management unit
DEP: Distributed energy prosumer
DSO: Distribution system operator

## Symbol

2025: Year 2025

## Units

TW: Tera Watt
Kg: Kilogram
kW: Kilo Watt
TWh: Tera Watt hour
GtCO_2_: Giga ton CO_2_
